# Autologous transplantation of adipose-derived stromal cells ameliorates ventilator-induced lung injury in rats

**DOI:** 10.1186/1479-5876-11-179

**Published:** 2013-07-26

**Authors:** Zuo Di Liang, Xiu Ru Yin, Da Sheng Cai, Heng Zhou, Ling Pei

**Affiliations:** 1 Anesthesiology Department, the First Hospital Affiliated at China Medical University, 155 Nanjing Bei Street, Shenyang 110001, China

**Keywords:** Cell therapy, Ventilator-induced lung injury, Alveolar fluid clearance, Na^+^ channel, Na,K-adenosine triphosphatase

## Abstract

**Background:**

Adipose-derived stromal cells (ADSCs) are a good alternative to multipotent stem cells for regenerative medicine. Low tidal volume (LVT) has proved to be an effective ventilation strategy. However, it is not known if ADSCs and LVT can protect against ventilator-induced lung injury (VILI). This study was aimed to determine the potential of ADSCs and LVT to repair following VILI and to elucidate the mechanisms responsible for this section.

**Methods:**

A total of 72 rats were randomly assigned into group I (sham group, n = 18), group II (1 h of high tidal volume-ventilated (HVT) 40 mL/kg to peak airway pressures of approximately 35 cm H_2_O and 100% oxygen, n = 18), group III (1 h of HVT followed by 6 h LVT 6 mL/kg to peak airway pressures of approximately 6 cm H_2_O and 100% oxygen, n = 18) and group IV (1 h of HVT followed by intravenous injection of 5 × 10^6^ ADSCs, n = 18). All animals were sacrificed 7 after the experiments lasted for 7 hours. Bronchoalveolar lavage fluid (BALF) was collected and lungs were harvested for analysis.

**Results:**

High tidal volume-ventilated (HVT) rats exhibited typical VILI features compared with sham rats. Lung edema, histological lung injury index, concentrations of total protein, total cell counts, number of neutrophils in bronchoalveolar lavage fluid (BALF), tumor necrosis factor-α, interleukin (IL)-1β, IL-6, IL-10 and transforming growth factor-β1 in BALF were significantly increased in HVT rats. Additionally, gene and protein levels of Na^+^ channel subunits, Na-K-ATPase pump activity and alveolar fluid clearance were significantly decreased in HVT rats. All these indices of VILI were significantly improved in rats treated with ADSCs. However, compared with ADSCs treatment, LVT strategy had little therapeutic effect in the present study.

**Conclusion:**

These results may provide valuable insights into the effects of ADSCs in acute lung injury.

## Introduction

Acute lung injury (ALI) and its more devastating form, acute respiratory distress syndrome (ARDS), are devastating clinical syndrome of the lung [1] and important causes of morbidity and mortality in critically ill patients [2]. Pathologically, ALI/ARDS are characterized by damage to the “pulmonary alveoli-capillary” barrier leading to the accumulation of protein-rich edema fluid in the alveolar space.

Active Na^+^ transport across the alveolar epithelium is a major determinant of alveolar fluid clearance (AFC) from the alveolar space into the interstitium and the pulmonary circulation. Na^+^ enters the cell via the Na^+^ channel (ENaC) located at the apical surface [3] and is subsequently pumped out of the cell by the Na,K-adenosine triphosphatase (Na,K-ATPase) on the basolateral side [4]. ENaC is a heterotrimer of three transmembrane subunits (α, β, and γ), which are able to reconstitute a functional channel [5]. α-ENaC gene knock-out mice which was unable to clear alveolar edema fluid died within 40 hours after birth [6]. β-ENaC and γ-ENaC gene were proved to influence the alveolar edema fluid absorption that was essential for AFC [7,8]. Therefore, the three subunits of ENaC play a pivotal role in AFC. Most patients with ALI/ARDS have impaired AFC [9] which is associated with poor prognosis [10]. Furthermore, up-regulation of ENaC and Na,K-ATPase is known to increase active Na^+^ transport, leading to a decrease in the severity of ALI [11] and improved survival in animals and humans with ALI/ARDS [12].

Patients with ALI/ARDS frequently require mechanical ventilation (MV) to decrease work of breathing and to maintain adequate gas exchange. However, high tidal volume (HVT) MV may result in deleterious physiologic and morphologic alterations [13]. MV can directly induce ALI (ventilator-induced lung injury, VILI) by damaging the alveolocapillary barrier [1,14], inducing proinflammatory cytokines [15]. Furthermore, MV can directly impair AFC accompanied by a significant decrease in activity of Na,K-ATPase and active Na^+^ transport [16].

A new therapeutic approach involves developing strategies that enhance lung repair following VILI. Accumulating evidence from studies on animal models and human pulmonary tissue has shown that mesenchymal stem cell (MSC) therapy potential to improve pulmonary function in various pulmonary diseases, including ALI [17]. The proposed mechanisms include inhibition of inflammatory reactions, immunomodulation, and repair of damaged epithelial cells. Bone marrow (BM)-derived stem cells have been the primary source of stem cells for tissue engineering applications [18]; however, isolation of BM-MSCs requires bone marrow aspiration, which is a moderate-risk procedure [19]. The greatest limitation of isolated MSCs from aspirated bone marrow is the low numbers of cells that can be obtained from the limited volume of marrow [20]. Recent studies have shown that subcutaneous adipose tissue has distinct advantages over other stem cell sources because of the ease of obtaining cells with minimal invasiveness, and the ability of these cells to be readily cultured to a sufficient number for autologous transplantation without the ethical issue of allografting. Moreover, it has been demonstrated that, ADSCs secrete significantly more bioactive factors than BM-derived stem cells, which may account for their superior anti-inflammatory and regeneration-enhancing properties [21].

The use of low tidal volumes during ventilation in patients with acute lung injury may reduce the release of inflammatory mediators [22]. Studies in animal models also demonstrated that low tidal volume ventilation protects both the alveolar epithelium and the endothelium in acid aspiration-induced ALI [14,23], suggesting a therapeutic benefit of this ventilation strategy in the management of ALI/ARDS. To date this low ventilation which is lung protective remains the only intervention with a confirmed benefit on clinical outcome and clearly demonstrates how studies on ALI/ARDS in organ and animal models have translated into a successful therapeutic strategy.

Currently, little is known regarding the effect of ADSCs and LVT in experimental models of ALI and pulmonary edema. Therefore, the aim of this study was to investigate and compare the effects of ADSCs and LVT on ENaC and Na,K-ATPase *in vivo.* We hypothesized that the administration of ADSCs and LVT might increase the activities of ENaC and Na,K-ATPase in an acute animal model of VILI, and might thus improve AFC.

## Materials and methods

Pathogen-free adult male Sprague–Dawley rats were obtained from the Laboratory Animal Center of China Medical University. All experimental animal procedures were performed in accordance with the Guide for the Care and Use of Laboratory Animals (National Institutes of Health (NIH) Guide publication no. 85–23, revised 1985, http://grants.nih.gov/grants/olaw/Guide-for-the-Care-and-Use-of-Laboratory-Animals.pdf and approved by the Ethics Review Committee for Animal Experimentation of the China Medical University.

### Isolation, culture and characterization of ADSCs

ADSCs were isolated from the inguinal fat pad by enzyme digestion and cultured as described previously with minor modifications [24]. Briefly, the adipose tissues were minced into small <1 mm^3^ fragments and incubated with 0.1% collagenase type I (Sigma-Aldrich, USA) in water bath under continuous shaking (37°C, 90 min) to digest the tissue. Collagenase was then neutralized by adding an equivalent DMEM/F12 (Gibco, USA) containing 10% fetal bovine serum (FBS; Gibco) to the tissue sample. The digested tissues were separated from the floating adipocytes by two centrifugations at 600 *g* for 5 min at room temperature. The mature adipose cells on the upper layer were removed and the pellet, as the stromal vascular fraction, was resuspended and filtered through a 40-μm filter. The fraction was then centrifuged for 5 min (50 *g*) to remove supernatant containing the cell debris. The cell pellets were plated in 25-cm^2^ culture flasks (Corning, USA) filled with 5 ml DMEM/F12 containing 15% FBS and 100 μg/ml penicillin/streptomycin. Cell cultures were maintained in a humidified tissue culture incubator (37°C, 5% CO_2_) and the medium was subsequently changed every 3 days for further cultivation. When ADSCs reached 90% confluence, the cells were passaged by 0.25% trypsin and 0.05% EDTA (Gibco) for analysis or transplantation. This study used ADSCs at their third passage.

To induce osteogenic differentiation, cells were cultured for 3 weeks in osteogenic medium (DMEM supplemented with 10% FBS, 10 mM β-glycerophosphate, 0.1 mM dexamethasone, and 50 mM ascorbic acid), as described previously [25]. Early mineralization was detected using Alizarin Red S. For adipogenic differentiation, cells were cultured in adipogenic differentiation medium (DMEM containing 10% FBS, 0.5 mM isobutylmethylxanthine, 200 μM indomethacin, 10 μM bovine insulin, and 1 μM dexamethasone), and Oil Red-O staining was performed after 21 days [26].

For phenotypic characterization, approximately 1 × 10^5^ cells were incubated for 30 min with monoclonal antibodies to CD29, CD105, CD90, CD45 and CD14 labeled with fluorescein isothiocyanate or phycoerythrin. Cells were analyzed using fluorescence-activated cell sorter (FACSCalibur, BD Biosciences, USA) and Cell Quest software.

### Induction of VILI and administration of ADSCs

Two weeks after surgery, Sprague–Dawley (body weights 260 ± 16 g) rats were anesthetized by intraperitoneal injection of pentobarbital 75 ml/kg (Sigma-Aldrich, USA). The rectal temperature of the rats was maintained within the range of 36.5–37.5°C throughout the procedure. Rats were placed supine and ventilated through a tracheotomy tube (16-gauge, Becton Dickinson, USA) with a volume-controlled ventilator (Model 683; Harvard Apparatus, South Natick, MA) at the following settings. A total of 72 animals were randomly assigned to four groups (n = 18 each). Group I: sham group; animals received no VILI or treatment and were kept under spontaneous breathing for the entire duration of the experiment. Group II: HVT; this experimental protocol has been reported previously [16]. Briefly, rats were ventilated for 60 min with HVT 40 mL/kg to peak airway pressures of 35 cm H_2_O and a respiratory rate of 40 breaths/min 100% oxygen. When high-stretch ventilation was discontinued, the animals were allowed to recover, and subsequently returned to their cages. Group III: HVT + low tidal volume (LVT); rats received HVT as in Group II, plus an additional low tidal volume of 6 mL/kg (LVT 6 h) to peak airway pressures of approximately 8 cm H_2_O and 100% oxygen. Group IV: HVT + ADSCs; rats received HVT as in Group II, plus autologous ADSCs (5 × 10^6^) were slowly infused approximately 5 min via a jugular venous cannula after 1 h of HVT 40 mL/kg. After ADSCs were infused, the animals were allowed to recover as in Group II. Anesthesia was maintained with repeated pentobarbital (500 μg/kg/h intravenous) and muscle relaxation was achieved with cisatracurium besylate 0.5 mg/kg. All animals were sacrificed 7 after the experiments lasted for 7 hours. Bronchoalveolar lavage fluid (BALF) was collected and lungs were obtained for analysis.

### Obtaining and processing bronchoalveolar lavage fluid (BALF)

Each animal was sacrificed by eyeball removal and blood letting. After exsanguination, the right main bronchus of rats in each group (n = 6) was tied with string at the right hilum. BALF was obtained from the left lung (n = 6) by using a 20-gauge angiocatheter ligated into the trachea and then flushing through it 3 mL of 0.9% NaCl three times. BALF recovery was always greater than 90% from each animal and was centrifuged at 1,200 *g* for 10 min at 4°C to remove cell debris. Total cell numbers per milliliter in the BALF were counted, and differential cell counts were performed. The supernatant was stored at -80°C for subsequent analysis. The concentrations of tumor necrosis factor (TNF)-α, interleukin (IL)-1β, IL-6, IL-10, transforming growth factor (TGF)-β1 and keratinocyte growth factor (KGF) in BALF fluid were determined using enzyme-linked immunosorbent assay (ELISA, R&D Systems, USA) kits. Total protein content of BAL supernatant was determined using a BCA protein assay kit (Pierce, USA).

### Lung wet/dry weight ratio

The right upper lobe of the same rats (n = 6) was ligated, excised, and weighed in a tared container immediately after exsanguination. The right upper lobe was then dried in an oven at 80°C until a constant weight was obtained and the wet-to-dry lung weight ratio was calculated.

### Lung histology

The right lower lung lobes of the same rats (n = 6) were resected and fixed in 4% paraformaldehyde (Sigma-Aldrich) for 24 h embedded in paraffin and stained with hematoxylin and eosin (H&E) staining kit (Beyotime Institute of Biotechnology, China) for microscopic observation. Two random tissue sections from four different lungs in each group were examined by a blinded investigator. Each subject was assessed according to a five-point scale: 0 = no injury; 1 = slight injury (25%); 2 = moderate injury (50%); 3 = severe injury (75%); and 4 = very severe injury (almost 100%).

### RNA extraction and real-time polymerase chain reaction (PCR) for α,β, and γ-ENaC

Total RNA from the residual right frozen tissues of the same rats (n = 6) was isolated using Trizol reagent. Quantitative real-time polymerase chain reaction (qPCR) was performed using SYBR Premix Ex Taq (Takara, Japan) in a total volume of 20 μl on a 7300 Real-Time PCR System (Takara, Japan): 95°C for 30 s, then 40 cycles of 95°C for 5 s, 60°C for 31 s. The sequences of the primer pairs were: α-ENaC forward, 5'-CATGCAAGGACTGGGGAAGG-3', reverse, 3'-TGGTCATGATCCTGCTGCTTAG-5'; β-ENaC forward, 5-AGAAGAAGGCCATGTGGTTCC-3', reverse, 3'-GCTCAGGTAGGTCTG- GATGAAG-5'; γ–ENaC forward, 5'-AGAAGAAGGCCATGTGGTTCC-3', reverse, 3'-GCTCAG- GTAGGTCTGGATGAAG-5'.

The comparative Ct method was used as described by Livak [27] for quantitation of gene expression. The Ct values of samples (group II–IV) and sham (group I) were normalized to the glyceraldehyde phosphate 3 dehydrogenase (GAPDH). The relative levels of gene expression were calculated as ∆Ct1 = Ct of ENaC – Ct of GAPDH (group II–IV) and ∆Ct2 = Ct of ENaC – Ct of GAPDH (group I), and the fold change of gene expression was calculated by the 2^-∆∆Ct^ method where ∆∆Ct = ∆Ct1–∆Ct2. Experiments were repeated in triplicate.

### Western blotting analysis

The right lung tissues proteins of other rats in each group (n = 6) were obtained with 1 ml of lysis buffer and 1 ml of extraction buffer using a protein extraction kit (Pierce) according to the manufacturer’ s instructions and stored at -80°C for analysis. Proteins were separated by 10% sodium dodecyl sulfate-polyacrylamide gel electrophoresis and transferred onto polyvinylidene fluoride membranes. After blocking with 5% nonfat dried milk in Tris-buffered (Sigma-Aldrich) saline containing 0.05% Tween 20 (Sigma-Aldrich), the membranes were incubated with primary antibodies α, β, γ-ENaC (1: 300 Santa Cruz Biotechnology, USA), and β-actin (1:500) overnight at 4°C, and then reacted with horseradish peroxidase-conjugated secondary antibody (1:5000) (Santa Cruz Biotechnology) at room temperature for 1 h. Three 10-min TBS-Tween washes were performed at 24°C after each incubation. Using a western blot enhanced chemiluminescence method [28], protein bands were visualized with a UVP Gel imaging system (Upland, CA, USA) and analyzed with Labworks software.

### Na,K-ATPase function

The residual left lung basolateral membrane proteins (BLMs) of the same rats (n = 6) were obtained from basolateral cell membranes isolated from the 1–2 mm of peripheral homogenizing lung tissue [29]. Na,K-ATPase activity was quantified by comparing the amount of inorganic phosphate (Pi) liberated from ATP over 1 h by 20 μg of BLMs in the absence or presence of the Na,K-ATPase inhibitor ouabain under conditions that maximize Na,K-ATPase activity (Vmax), as described previously [30]. Conditions used maximize Na,K-ATPase activity (Vmax) to produce an index of functional, membrane-bound receptor number.

### AFC measurement

AFC from the alveolar airspace was performed by the residual rats in each group as described previously [31]. Clearance was expressed by the change in concentration of Evan’s blue-tagged albumin in an iso-osmolar alveolar instillate placed into the alveolar airspace over a 30-minute period of measurement (n = 6). AFC was calculated as follows: AFC = (1-C_0_/C_30_), where C_0_, is the protein concentration of the instillate before instillation, and C_30_ is the protein concentration of the sample obtained at the end of 30-min of mechanical ventilation. Clearance was expressed as a percentage of total instilled volume cleared in 30 min.

### Statistical analysis

All data were described as mean ± SEM. Statistical analysis was performed using SPSS16.0 software. Results were compared by one-way ANOVA followed by Student-Newman-Keuls test, or by one-way ANOVA on ranks followed by Bonferroni-Dunn test. Statistical significance was confirmed at a two-tailed p value of <0.05.

## Results

### Morphology and characterization of ADSCs

**Figure 1:**
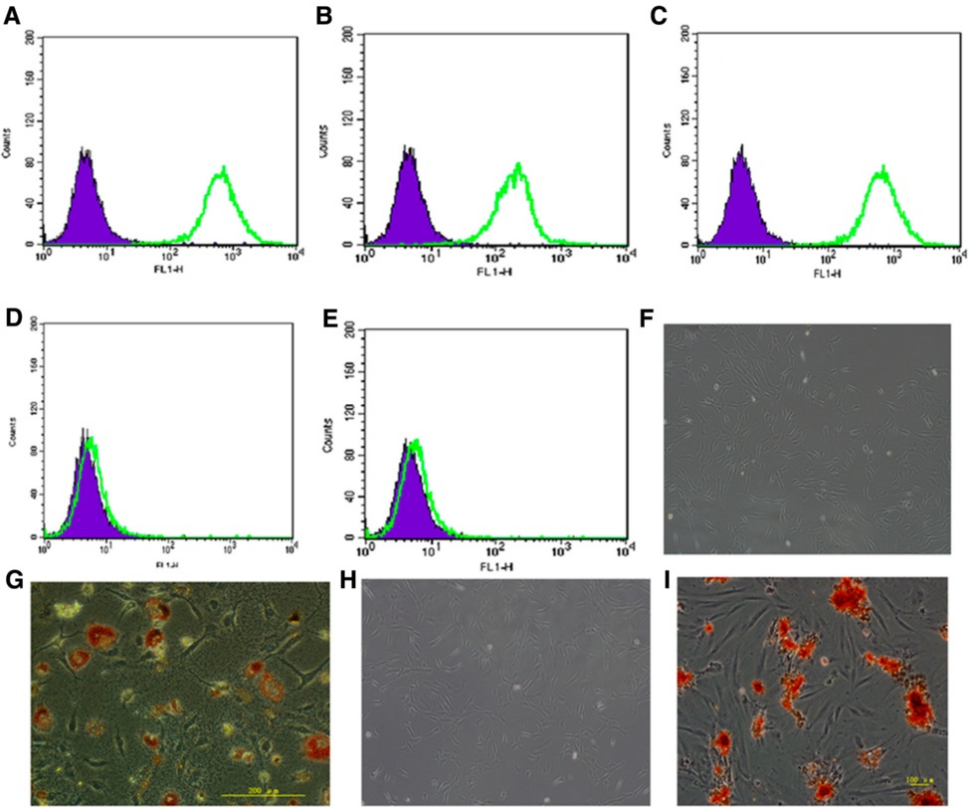
**Surface markers and induced differentiation of ADSCs at passage 3. (A)** CD29. **(B)** CD105. **(C)** CD90. **(D)** CD45. **(E)** CD14. **(F)** Oil Red-O staining of control micrograph. **(G)** Oil Red-O staining after adipogenic induction. **(H)** Alizarin red staining of control micrograph. **(I)** Alizarin red staining after osteogenic induction.

ADSCs isolated from inguinal adipose had the ability to self-renew and adhere to plastic, and expanded in culture without losing differentiation potential. Flow cytometric analysis demonstrated that ADSCs were positive for CD29, CD90, and CD105, but negative for CD45 and CD14 (Figure 1A–E) as reported previously [32]. These cells could be induced to differentiate into mature adipocytes, which were confirmed by intracellular lipid droplets after Oil Red-O staining (Figure 1G). ADSCs also differentiated into osteoblasts and had the capacity to deposit calcium, as demonstrated by alizarin red staining (Figure 1I). In the control micrographs for adipogenesis and osteogenesis, these changes were not observed (Figure 1F and 1H).

The above experimental results indicated that we have successfully isolated mesenchymal stem cells from adipose tissue.

### ADSCs attenuated lung injury in HVT-induced acute lung injury

**Figure 2:**
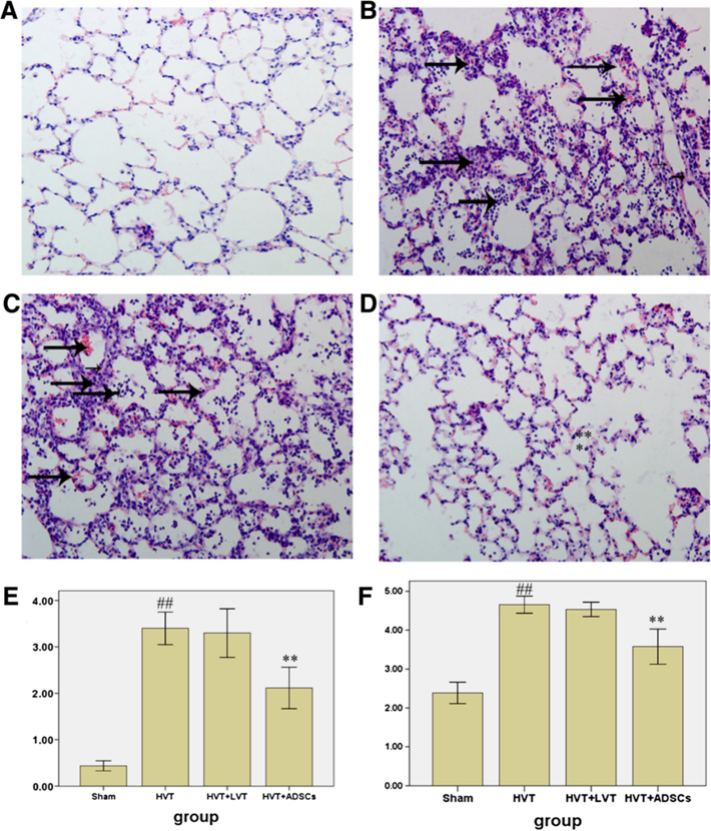
**Effect of ADSCs on lung histopathology. (A)** Sham group. **(B)** HVT group **(C)** HVT + LVT group. **(D)** HVT + ADSCs group. **(E)** Lung injury score. **(F)** Lung wet/dry. Data are presented as mean ± SEM. ## p < 0.01 vs sham group. **p < 0.01 vs HVT group. n = 6 animals in each group and each assay was repeated 3 times.

Before sacrificed, all animals survived in each group. H&E-stained lung sections showed normal lung tissue structures in the sham group (Figure 2A). HVT induced prominent lesions, such as interlobular septal thickening, interstitial inflammation, hemorrhage, and infiltration of neutrophils into alveolar spaces (Figure 2B). These were rarely improved in the LVT group (Figure 2C). In contrast, the alveolar architecture was well preserved and histological changes were attenuated in the ADSC-treated group (Figure 2D). The computerized lung injury score provided consistent results (p < 0.01 for sham vs. HVT and HVT vs. HVT + ADSCs, Figure 2E). Lung edema assessed by the wet/dry weight ratio method was significantly greater in rats subjected to over-ventilation compared with controls. However, treatment with ADSCs considerably reduced edema (p < 0.01, Figure 2F).

### Effect of ADSCs and LVT on total protein, neutrophil infiltration, cytokines and chemokines in VILI

**Figure 3:**
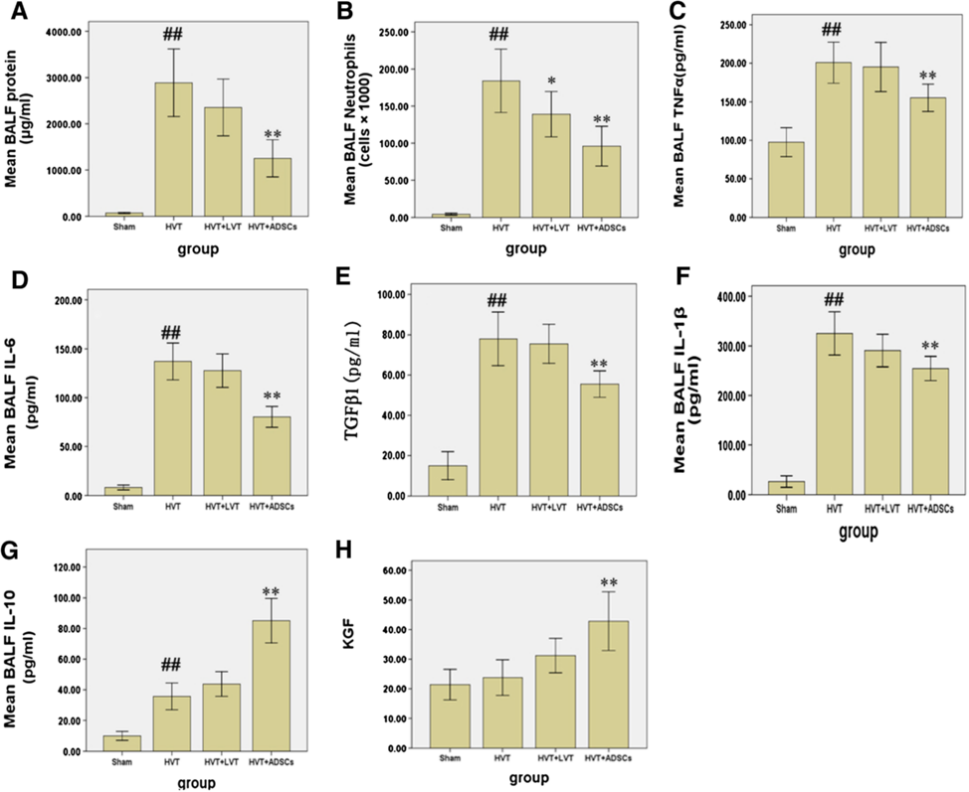
**Effects of ADSCs on total protein, neutrophil infiltration, cytokines and chemokines response in BALF. (A)** BALF total protein. **(B)** neutrophil counts **(C)** TNF-α concentration **(D)** IL-6 concentration **(E)** TGF-β1 concentration **(F)** IL-β1 concentration **(G)** IL-10 concentration **(H)** KGF concentration. Data are presented as mean ± SEM. ## p < 0.01 vs sham group. *p < 0.05 vs HVT group. **p < 0.01 vs HVT group. n = 6 animals in each group and each assay was repeated 3 times.

ADSCs significantly reduced VILI-induced increases in TNF-α, IL-6, IL-1β, protein level, TGF-β1, total cell counts, and neutrophil counts in BALF (p < 0.01, Figure 3A–F). Furthermore, alveolar concentrations of IL-10 (Figure 3G) and KGF (Figure 3H), epithelial-specific growth factor and anti-inflammatory cytokines were remarkably increased in the ADSC group. In contrast, although the levels were ameliorated in LVT group, there was no significant difference except in the case of neutrophil counts (p < 0.05, Figure 3B).

### Effects of ADSCs on gene and protein expression of ENaC subunits

**Figure 4:**
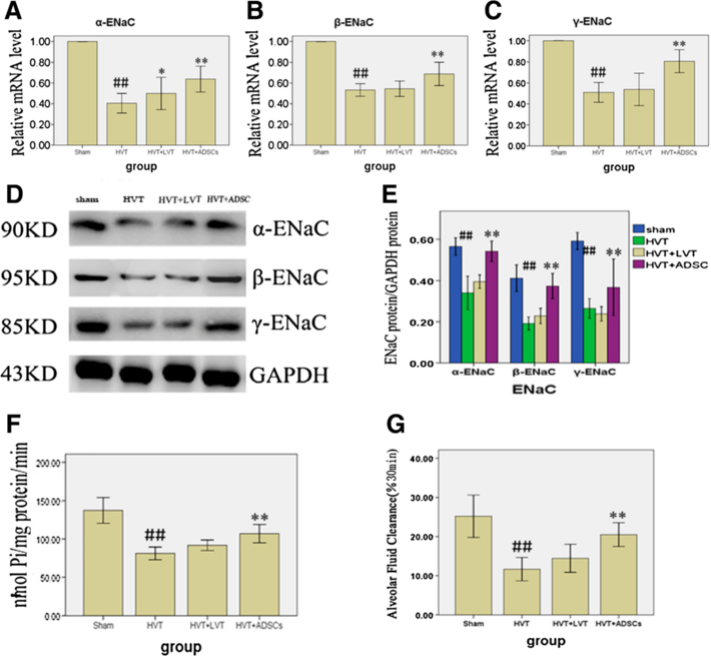
**Effects of ADSCs on gene and protein expression of ENaC subunits. (A–C)** α-, β- and γ-ENaC relative mRNA level. **(D–E)** α-, β- and γ-ENaC protein expression. **(F)** Na,K-ATPase activity. **(G)** Alveolar Fluid Clearance. Data are presented as mean ± SEM. ## p < 0.01 vs sham group. *p < 0.05 vs HVT group. **p < 0.01 vs HVT group. n = 6 animals in each group and each assay was repeated three times.

mRNA and protein expression levels of α-, β- and γ-ENaC in rat lung were significantly lower in the HVT group, compared with the sham group (p < 0.01) but the mRNA and protein expression levels of the three ENaC subunits were significantly increased following administration of ADSCs, compared with the HVT and LVT groups (p < 0.01, Figure 4A–E). α-ENaC mRNA levels were significantly increased in the LVT group, compared with the HVT group (p < 0.05, Figure 4A).

### Effects of ADSCs on distal lung Na-K-ATPase pump activity

Na-K-ATPase pump activity in peripheral lung tissue was examined 6 h after VILI and was decreased compared with that in the control group. However, the activity was significantly improved in the ADSCs group, to values very close to those in controls (p < 0.01, Figure 4F).

### Effects of ADSCs on alveolar fluid clearance

To examine the physiological significance of these changes in mRNA and protein levels, we measured AFC rates in our HVT model. Consistent with the results of other studies, basal mean AFC was significantly decreased in the HVT group compared with the sham group and infusion of ADSCs 6 h following the induction of ALI significantly increased AFC rates (p < 0.01, Figure 4G).

## Discussion

This study demonstrated that autologous transplantation of ADSCs significantly ameliorated VILI through the inhibition of several proinflammatory cytokines and by increasing the lung’s ability to clear edema by up-regulation of ENaC gene expression and Na,K-ATPase activity in the lung.

VILI can cause capillary stress fracture of the alveolocapillary barrier and the release of proinflammatory mediators which not only increase lung permeability to small and large solutes but also decrease active Na^+^ transport and AFC in association with down-regulation of alveolar epithelial Na,K-ATPase functioning [16,33]. The current study used a common rat model of VILI, with specific magnitude of overstretch (40 mL/kg) and 1-h duration of injurious mechanical stimulation [16]. The rats subjected to lung over-distension exhibited the expected pattern of VILI namely lung inflammation, lung edema and considerable histopathological changes [34]. Moreover, in addition to the decline in Na-K ATPase activity, ENaC gene and protein expression levels were also significantly decreased in rats subjected to HVT.

Treatment with ADSCs significantly reduced the development of VILI in rats subjected to HVT. Our results indicated that all the variables assessed in the animals treated with ADSCs tended to recover towards the control values, i.e. edema and histological index of lung injury, neutrophils counts and biomolecule concentrations in the BALF. Taken together, these data provide clear evidence for amelioration of the increased alveolocapillary membrane permeability and the local inflammatory process induced by HVT. The effectiveness of ADSCs in minimizing VILI was in agreement with the results of other studies, where the protective effects of adult stem cells in VILI were investigated using bone marrow-derived mesenchymal cells [35,36]. Our results demonstrated that ADSCs also played a therapeutic role in VILI by improving AFC through increasing ENaC and Na,K-ATPase activity.

Investigators have reported that BALF in ALI contains high levels of several pro-inflammatory cytokines, including IL-1β, IL-6, IL-8, TNFα and TGFβ1 [37-39]. Several of these cytokines have been shown to have opposing effects on sodium transport and AFC. For example, TNFα decreased the expression of ENaC (α-, β-, γ-subunits) mRNA and protein levels [40]. Similarly, IL-1β [41] and TGFβ1 [42] decreased the expression of α-ENaC mRNA and protein levels. However, these cytokines were significantly reduced in the ADSC group in the present study. The potential mechanism for the therapeutic effects may be the immunomodulatory properties of MSCs which participates in down-regulation of inflammatory reaction [43]. Interestingly, the present study demonstrated notably increased pulmonary expressions of anti-inflammatory IL-10 in animals with ADSCs therapy. Another possible mechanism of MSCs may be the production of several epithelial-specific growth factors, specifically KGF. A previous report demonstrated that MSCs restored AFC via a KGF-dependent mechanism in an *ex vivo* perfused human lung endotoxin-induced injury [44]. Alveolar fluid transport in rat lung was partly improved by the secretion of KGF, which up-regulated α-ENaC gene expression [45] and Na,K ATPase activity [46]. The current results also showed that ADSCs produced substantial quantities of KGF in lung tissue. Therefore, we speculated that ADSCs could be responsible for inhibiting cytokine expression and reducing edema. However, further research in this field should be made and focus on elucidating the basic mechanisms responsible for the beneficial effects of ADSC, as well as determining the practical issues involved in producing a cell-based therapy for patients. In the process, a novel therapy for ALI/ARDS might emerge.

The mortality of patients with ALI treated with the LVT strategy was by reduced 9% compared with HVT-treated patients [47]. Subsequently, several pathogenesis studies have demonstrated that LVT reduces plasma levels of surfactant protein D, a product of alveolar epithelial type II cells and reduces the levels of inflammatory mediators in the plasma (IL-6 and IL-8, and TNF receptors 1 and 2) [22]. Although this lung-protective approach has proved to be an effective barrier and anti-inflammatory strategy for patients with ALI, LVT strategy had little therapeutic effect compared with ADSCs treatment in the present study. However, it is possible that we analyzed our results at an earlier time point.

Although the experimental design was similar to that of conventional studies using rodent VILI models and ADSC infusion, there were some limitations associated with the current study, because not all the possible ranges of variables were investigated. First, we did not provide control for the effects of ADSCs on unventilated, LVT 7 h alone and LVT treated at 0 time with ADSCs. However, our found that LVT did not aggravate injury and ADSCs can alleviate injury. Therefore, it would be expected that these influence on these animals would be limited. Second, although the proposed mechanism may serve as a scaffold outlining the possible relationships among our study parameters, the exact mechanisms underlying the observed improvement in VILI through ADSCs administration are likely to be more complex and possibly involve multiple compensatory routes. Lastly, solid cause-and-effect relationships underlying the exact mechanisms remain to be elucidated. In conclusion, we have shown that autologous transplantation of ADSCs has a better therapeutic effect than LVT and can be used to alleviate lung injury and restore lung fluid balance after VILI by increasing ENaC gene expression and Na,K-ATPase activity. The possible mechanism responsible for this beneficial effect on AFC is partly due to the secretion of paracrine soluble factors such as KGF by the ADSCs and their anti-inflammatory properties.

## Competing interests

The authors declare that they have no competing interests.

## Authors’ contributions

ZDL and LP participated in the design of the study, data acquisition and analysis as well as drafting the manuscript. XRY, DSC, and HZ were responsible for the laboratory assay and participated in the sequence alignment. All authors read and approved the final manuscript.
